# 胸腔积液沉淀物在恶性胸腔积液诊断中的价值

**DOI:** 10.3779/j.issn.1009-3419.2015.10.09

**Published:** 2015-10-20

**Affiliations:** 300052 天津，天津医科大学总医院肿瘤科 Department of Medical Oncology, Tianjin Medical University General Hospital, Tianjin 300052, China

**Keywords:** 恶性胸腔积液, 沉淀物, 诊断价值, Malignant pleural effusion, Cell block, Diagnostic value

## Abstract

**背景与目的**  恶性胸腔积液(malignant pleural effusion, MPE)是由原发于胸膜的恶性肿瘤或者是转移至胸膜的恶性肿瘤造成的胸腔积液。对于不明原因的单侧胸腔积液, 首要任务是排除或者是确诊恶性胸腔积液。胸腔积液沉淀物是将送检胸腔积液细胞学剩余的胸腔积液进行离心或者是自然静置所获得的细胞块。此技术具有操作简单、有创性小、重复性高、对恶性胸腔积液的诊断率相对较高等特点, 在恶性胸腔积液的诊断、治疗等方面起着重要的作用。本文主要从沉淀物的制作方法、免疫组织化学染色检查的鉴别诊断价值、沉淀物的诊断优势及沉淀物行基因检测的临床应用价值等方面来论述胸腔积液沉淀物对恶性胸腔积液的诊断价值。

## Abstract (en)

Malignant pleural effusion (MPE) is due tumor which arises from the mesothelium or metastases from tumor origniating other sites.In large, for undiagnosed unilateral pleural effusions, the most frequent and important diagnosis to be established or excluded is malignancy.Cell block is prepared from residual fluid which is centrifuged or is naturally sedimenting to obtain clots at the bottom of the container.The cell block technique is simple, relatively non-invasive, reproducible and has a high yield for malignant plerual effusion.It plays an important role in the diagnosis, guiding the treatment of maligant pleural effusion.Herein, we summarize the technologys which make the cell block, the differential diagnostic value when multiple sections of the cell block are processed for immunhistochemistry, advantages in the diagnosis of malignant pleural effusion, the clinical value of gene screening in cell block.The aim of this article is to discuss the value of cell block in diagnosis of maligant pleural effusion.

恶性胸腔积液的产生, 预示着恶性肿瘤的进展、生存期的缩短、生活质量的下降。恶性胸腔积液的生存期与恶性肿瘤的原发部位、分期、病理分型等密切相关, 平均中位生存期仅为3个月-12个月^[1]^。胸腔积液中找到肿瘤细胞或者是胸膜活检组织中观察到恶性肿瘤的病理变化是诊断恶性胸腔积液的金标准^[2]^。胸腔积液细胞涂片是诊断恶性胸腔积液的常规手段, 其诊断率约为60%左右^[3]^。与细胞涂片相比, 胸腔积液沉淀物通过包埋切片进一步行常规染色、免疫组织化学染色等, 不仅可以提高恶性胸腔积液的诊断率, 而且可以进一步明确恶性胸腔积液的原发病灶^[4]^。与胸腔镜等有创检查相比, 此项技术操作方便、有创性小, 可重复性高, 对于无法获得原发组织标本的患者, 不仅可以获得病理学的诊断, 而且可以进行相关基因的检测, 指导靶向治疗^[5]^。因此胸腔积液沉淀物对恶性胸腔积液的诊断具有独特的优势。

## 1 胸腔积液沉淀物的制备

胸腔积液沉淀物的制备并没有统一的标准方法, 不同的医疗机构, 制备方式不同。文献报道涉及到的方法有:细胞色素氧化酶凝血酶法(cytolyt-prefixed thrombin clot, CTC)、琼脂凝胶法(HistoGel, HG)、血浆凝血酶法(plasma thrombin, PT)、促凝血酶原激酶-血浆法(thromboplastin-plasma, TP)^[6, 7]^、过夜沉淀法、离心沉淀法^[8]^、反滤层沉积法、清蛋白法、简单沉降法^[9]^等。沉淀物切片制作人员的技术水平、切片中具有诊断意义的细胞的含量、染色后细胞形态学的表现、免疫组化非特异性背景染色的干扰程度、阅片病理学家的专业知识等因素, 均可影响沉淀物的诊断价值。不同的制作方法所需要的制作过程、消耗的时间、花费是不同的。Kulkarni等^[7]^采用TP法制备沉淀物, 结果显示, TP法能够保留更多具有诊断意义的细胞, 这些细胞具有相对完整的细胞结构、细胞形态学和细胞分布, 更利于鉴别诊断。需要指出的是, 目前还没有公认的最佳沉淀物的制备方法。

## 2 免疫组织化学染色的鉴别诊断价值

免疫组织化学染色是鉴别诊断不明病因胸腔积液的重要辅助手段。细胞涂片及沉淀物切片均可行免疫组织化学染色, 与细胞涂片相比, 沉淀物切片能够明显降低细胞的收缩、抗原的丢失, 减少背景染色等影响鉴定染色结果的因素; 另外, 沉淀物切片中的细胞形态与组织病理切片中的细胞形态相似, 并且保持着恶性细胞的组织排列方式, 免疫指标的阳性定位在沉淀物切片与组织学标本是一致的; 上述优点使沉淀物切片较细胞涂片更适合行免疫组织化学染色^[10]^。

常规染色一般可以明确细胞的良恶性, 但反应增生性间皮细胞、恶性间皮瘤细胞及腺癌细胞的细胞形态学有一定的相似性, 仅仅依靠细胞形态学, 不易区分上述细胞。联合应用不同抗体的免疫组织化学染色, 对上述三种细胞的鉴别诊断具有较高的敏感性和特异性^[11]^。例如:D2-40、calretinin、MOC-31抗体组合可用于鉴别恶性间皮瘤和转移性腺癌细胞^[12]^。E-cadherin和fibronectin抗体组合可用于鉴别转移性腺癌细胞和反应性间皮细胞^[13]^。转移性腺癌是恶性胸腔积液中最常见的病理类型, 不同来源的腺癌细胞的细胞形态学有一定的差异, 但仅仅依靠形态学差异很难确定恶性肿瘤的来源, 可结合病史、体格检查、影像学检查等, 选择组织特异性抗体的免疫组织化学染色以明确恶性胸腔积液的原发部位。临床上可用甲状腺转录因子-1(thyroid transcription factor 1, TTF-1)和新天冬氨酸蛋白酶A (noval aspartic proteinase of the pepsin family A, napsin A)抗体组合鉴别转移性肺腺癌^[14]^; 人表皮生长因子受体2(human epidermal growth factor receptor-2, Her2)、雌激素受体(estrogen receptor, ER)、孕激素受体(progesterone receptor, PR)抗体组合鉴别转移性乳腺癌^[15]^; 配对盒基因8(paired box gene 8, PAX8)和Wilms瘤基因(Wilms tumor 1, WT1)抗体组合鉴别转移性卵巢腺癌^[16]^。需要指出的是, 目前, 没有一种用于鉴别胸腔积液的良恶性或者原发部位的抗体具有100%的敏感性和特异性, 故需要用一系列的抗体组合来鉴定。一些学者正致力于研究和寻找能明确不同性质和来源的胸腔积液的“黄金抗体组合”。Su等^[17]^实验结果表明, E-cadherin、癌胚抗原(carcinoembryonic antigen, CEA)、calretinin和thrombomodulin抗体的组合可以用于腺癌和恶性间皮瘤/反应性间皮细胞的鉴别; EMA和Des可以用于恶性间皮瘤和反应性间皮细胞的鉴别。此外, Kim等^[18]^报道, 胰岛素样生长因子Ⅱ信使RNA结合蛋白-3(insulin-like growth factor-Ⅱ mRNA-binding protein 3, IMP3)不仅可用于鉴别转移性腺癌与增生性间皮细胞, 而且是转移性胃腺癌的潜在的预后指标。

既往有恶性肿瘤病史的患者, 出现了恶性胸腔积液, 也可能是由新发的恶性肿瘤引起, 通过免疫组织化学染色, 可以明确潜在的恶性肿瘤的部位^[19]^。此外, 利用免疫组织化学染色可以证实一些少见的引起胸腔积液的病因。Teresa等^[20]^报道, 通过沉淀物的免疫组织化学染色确诊肾嫌色细胞癌引起的恶性胸腔积液。

## 3 胸腔积液沉淀物的诊断优势

指南推荐同时送检胸腔积液细胞涂片及沉淀物, 以提高恶性胸腔积液的诊断率^[3]^。与单纯的细胞涂片相比, 沉淀物可以使恶性胸腔积液的诊断率提高10%-15%^[21, 22]^。Shivakumarswamy等^[22]^对60例原因待查的胸腔积液患者同时送检细胞涂片及沉淀物切片检查, 结果显示, 切片比细胞涂片多确诊了9例恶性胸腔积液, 恶性胸腔积液的诊断率提高了15%。Ghosh等^[4]^收集60例以胸腔积液为首发临床症状的患者, 结合临床资料, 均不排除恶性胸腔积液, 采用胸腔积液细胞涂片、沉淀物、支气管镜、胸膜活检术、胸腔镜等进一步明确诊断, 56例患者最终确诊为恶性胸腔积液, 其中根据沉淀物的病理结果确诊了46例, 而细胞涂片仅确诊了22例。Koksal等^[23]^研究发现, 与细胞涂片相比, 沉淀物不仅可以提高肺癌引起的恶性胸腔积液的检出率, 而且可增加癌组织类型的确诊率。

## 4 沉淀物在基因突变检测中的价值

胸腔积液可以是肺腺癌首发的临床表现, 也可以在进展期的肺腺癌中出现, 其主要原因为恶性肿瘤胸膜转移。表皮生长因子受体酪氨酸激酶抑制剂(epidermal growth factor receptor-tyrosine kinase inhibitors, EGFR-TKIs)对于具有*EGFR*基因突变的肺腺癌有很高的有效性, 相反, 对于无*EGFR*基因突变的肺腺癌效果很差或者无效。因此, *EGFR*基因突变的结果对于肺腺癌的靶向治疗是至关重要的^[24]^。通过胸腔镜或者经皮肺活检获得的胸膜组织或者肺组织是做*EGFR*基因突变检测的理想标本。但是, 对于某些肺腺癌伴恶性胸腔积液的患者, 由于主客观原因, 可能无法获得肺组织标本。由于胸腔积液很容易获得, 有研究者使用胸腔积液对*EGFR*基因突变检测的敏感性、特异性和实用性进行了研究^[25]^。Liu等^[5]^通过突变特异性扩增系统(amplified refractory mutation system, ARMS)测定胸腔积液(胸腔积液上清液和沉淀物)*EGFR*基因突变, 结果显示, 胸腔积液标本和转移性胸膜肿瘤组织的*EGFR*基因突变状态高度一致, 提示胸腔积液可以作为肿瘤组织的替代物行基因检测。Liu等^[26]^通过Sanger测序法和ARMS法, 测定胸腔积液和肿瘤组织的*EGFR*基因突变, 结果显示:与ARMS法相比, Sanger测序法测定肿瘤组织*EGFR*突变的敏感性为81.8%, 沉淀物*EGFR*突变的的敏感性为40%, ARMS法更适合测定沉淀物的*EGFR*突变。对于切片中细胞含量不足以行分子检测的患者, 可以行突变特异性抗体的免疫组织化学染色以明确基因突变的状态^[27, 28]^。Tsai等^[28]^利用免疫特异性抗体来检测沉淀物的L858R和delE746-A750的突变, 并给予突变阳性的患者EGFR-TKIs靶向治疗以明确治疗的有效率, 结果显示免疫特异性抗体证实的突变阳性的患者对EGFR-TKIs治疗的反应率为67%。免疫组织化学法操作简单, 但敏感性和特异性均低于分子检测技术^[26]^。此外, 也可使用胸腔积液沉淀物进行间变性淋巴瘤激酶(anaplastic lymphoma kinase, *ALK*)基因的检测^[29]^。目前关于沉淀物行基因检测相关研究的标本数目尚少, 仍需要大量的临床实践来进一步证实上述观点。

## 5 结论

胸腔积液沉淀物取材方便、有创性小、相关检查的可重复性高, 联合常规染色、特殊染色、免疫组织化学染色等, 可提高恶性胸腔积液的诊断率、确定恶性胸腔积液的原发病灶。对于无法获得组织标本的非小细胞肺癌患者, 沉淀物代替组织标本行相关基因的检测, 可指导靶向治疗。
